# Carbon Monitor, a near-real-time daily dataset of global CO_2_ emission from fossil fuel and cement production

**DOI:** 10.1038/s41597-020-00708-7

**Published:** 2020-11-09

**Authors:** Zhu Liu, Philippe Ciais, Zhu Deng, Steven J. Davis, Bo Zheng, Yilong Wang, Duo Cui, Biqing Zhu, Xinyu Dou, Piyu Ke, Taochun Sun, Rui Guo, Haiwang Zhong, Olivier Boucher, François-Marie Bréon, Chenxi Lu, Runtao Guo, Jinjun Xue, Eulalie Boucher, Katsumasa Tanaka, Frédéric Chevallier

**Affiliations:** 1 Department of Earth System Science, Tsinghua University, Beijing, 100084 China; 2 Laboratoire des Sciences du Climat et de l’Environnement (LSCE/IPSL), CEA-CNRS-UVSQ, Univ Paris-Saclay, Gif-sur-Yvette, France; 3 Department of Earth System Science, University of California, Irvine, 3232 Croul Hall, Irvine, CA 92697-3100 USA; 4 Key Laboratory of Land Surface Pattern and Simulation, Institute of Geographical Sciences and Natural Resources Research, Chinese Academy of Sciences, Beijing, China; 5 Department of Electrical Engineering, Tsinghua University, Beijing, 100084 China; 6 Institute Pierre-Simon Laplace, Sorbonne Université/CNRS, Paris, France; 7 School of Mathematical School, Tsinghua University, Beijing, 100084 China; 8 Center of Hubei Cooperative Innovation for Emissions Trading System, Wuhan, China; 9 Faculty of Management and Economics, Kunming University of Science and Technology, Kunming, China; 10 Economic Research Centre of Nagoya University, Furo-cho, Chikusa-ku, Nagoya, Japan; 11 Université Paris-Dauphine, PSL Paris, France; 12 Center for Global Environmental Research, National Institute for Environmental Studies, Tsukuba, Japan

**Keywords:** Climate-change policy, Energy and society, Climate-change mitigation

## Abstract

We constructed a near-real-time daily CO_2_ emission dataset, the Carbon Monitor, to monitor the variations in CO_2_ emissions from fossil fuel combustion and cement production since January 1, 2019, at the national level, with near-global coverage on a daily basis and the potential to be frequently updated. Daily CO_2_ emissions are estimated from a diverse range of activity data, including the hourly to daily electrical power generation data of 31 countries, monthly production data and production indices of industry processes of 62 countries/regions, and daily mobility data and mobility indices for the ground transportation of 416 cities worldwide. Individual flight location data and monthly data were utilized for aviation and maritime transportation sector estimates. In addition, monthly fuel consumption data corrected for the daily air temperature of 206 countries were used to estimate the emissions from commercial and residential buildings. This Carbon Monitor dataset manifests the dynamic nature of CO_2_ emissions through daily, weekly and seasonal variations as influenced by workdays and holidays, as well as by the unfolding impacts of the COVID-19 pandemic. The Carbon Monitor near-real-time CO_2_ emission dataset shows a 8.8% decline in CO_2_ emissions globally from January 1^st^ to June 30^th^ in 2020 when compared with the same period in 2019 and detects a regrowth of CO_2_ emissions by late April, which is mainly attributed to the recovery of economic activities in China and a partial easing of lockdowns in other countries. This daily updated CO_2_ emission dataset could offer a range of opportunities for related scientific research and policy making.

## Abstract (summary)

**Table:** 

Measurement(s)	carbon dioxide emission
Technology Type(s)	computational modeling technique
Factor Type(s)	geographic location • sector • temporal interval
Sample Characteristic - Environment	climate system
Sample Characteristic - Location	global

Machine-accessible metadata file describing the reported data: 10.6084/m9.figshare.12994058

## Background & Summary

The main cause of global climate change is the excessive anthropogenic emission of CO_2_ to the atmosphere from geological carbon reservoirs, the combustion of fossil fuel and cement production. Dynamic information on fossil fuel-related CO_2_ emissions is critical for understanding the impacts of different human activities and their variability on driving climate change. Emissions with high-temporal resolution help monitoring changes in emissions from activity data, during the COVID-19 and for likely weaker signals thereafter. Furthermore, the combustion processes of fossil fuel also emit short-lived pollutants such as SO_2_, NO_2_ and CO, and capturing these data would also allow a more accurate quantification and better understanding of air quality changes^1,2^. Estimates of CO_2_ emissions from fossil fuel combustion and cement production^2–7^ are based on both activity data (e.g., the amount of fuel burnt or energy produced) and emissions factors (see Methods)^8^. The sources of these data are mainly national energy statistics, although a number of databases, such as CDIAC, BP, EDGAR, IEA and GCP, also produce and compile estimates for different groups of countries or for all countries^1,9–11^. Fossil fuel-related CO_2_ emissions are usually reported on an annual basis but the data lag by at least one year.

The uncertainty associated with CO_2_ emissions from burning fossil fuel and producing cement is small when considering large emitters or the global totals: smaller than that of co-emitted combustion-related pollutants, for which uncertain technological factors influence the ratio of emitted amounts to fossil fuel burnt^12–14^. The uncertainty of global carbon emissions from fossil fuel burning and cement production varies between ±6% and ±10%^2,6,15,16^ (±2σ), and it is attributed to both the activity data and the emissions factors. For the activity data, the amount of fuel burnt is captured by energy production and consumption statistics; hence, the uncertainties are introduced by errors and inconsistencies in the reported figures from different sources. For the emissions factors, the different fuel types, quality and combustion efficiency together contribute to the overall uncertainty. For example, coal used in China is of variable quality, and its emission factors, both before cleaning (raw coal) and after (cleaned coal), vary significantly, which was found to cause a 15% uncertainty range for CO_2_ emissions^17^. On the other hand, there is very limited temporal change in emission factors. For example, the annual difference in emission factors for coal consumption was within 2% globally^17–20^, while the variation in emission factors for oil and gas was found to be much smaller.

Given that the uncertainty of CO_2_ emissions from fossil fuel burning and cement production is generally below ±10%^9,21,22^ and the annual difference in emission factors is less than 2%^17^, the CO_2_ emissions can be estimated directly by estimating the absolute amount of and the relative change in activity over time. This method has been widely used for scientific products that update recent changes in CO_2_ emissions estimates^1,23–25^, given that official and comprehensive CO_2_ national inventories reported by countries to the UNFCCC become available with a lag of two years for Annex-I countries and several years for non-Annex-I countries^4^. As such, a higher spatial, temporal and sectoral resolution of CO_2_ emission inventories beyond annual and national levels can be obtained by using spatial, temporal and sectoral data to disaggregate annual national emissions^8,13,25,26^. The level of granularity in spatially explicit dynamic emission inventories depends on the available data, such as the location and operations of sources^25^ (i.e., power generation for a certain plant), regional statistics of energy use (i.e., monthly fuel consumption)^8,26^, and knowledge about proxies for the distribution of emissions such as gridded population density, night lights, urban forms and GDP data^8,13,25,26^.

Gaining from past experiences in constructing annual inventories and newly compiled activity data, we present in this study a novel daily dataset of CO_2_ emissions from fossil fuel burning and cement production at the national level. The countries/regions include China, India, the US, EU27 & UK, Russia, Japan, Brazil, and the rest of the world (ROW), as well as the emissions from international bunkers. This dataset, known as Carbon Monitor (data available at https://carbonmonitor.org/), is separated into several key emission sectors: power sector (39% of total emissions), industrial production (28%), ground transport (19%), air transport (3%), ship transport (2%), and residential consumption (10%). For the first time, daily emissions estimates are produced for these six sectors based on dynamically and regularly updated activity data. This is made possible by the availability of recent activity data such as hourly electrical power generation, traffic indices, airplane locations and natural gas distribution, with the assumption that the daily variation in emissions is driven by the activity data and that the contribution from emission factors is negligible, as they evolve at longer time scales, e.g., from policy implementation and technology shifts.

**Fig. 1:**
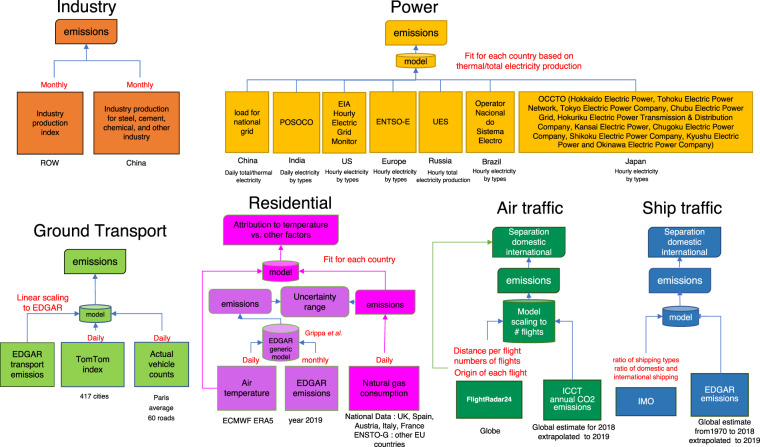
Framework for data processing.

The framework of this study is illustrated in Fig. 1. We calculated national CO_2_ emissions and international aviation and shipping emissions since January 1, 2019, drawing on hourly datasets of electric power production and the associated CO_2_ emissions in 31 countries (thus including the substantial variations in carbon intensity associated with the variable mix of electricity production), daily vehicle traffic indices in 416 cities worldwide, monthly production data for cement, steel and other energy intensive industrial products in 62 countries/regions, daily maritime and aircraft transportation activity data, and previous-year fuel use data corrected for air temperature for residential and commercial buildings, covering over 70% of global power and industry emissions, 85% of ground transportation emissions, 100% of residential and international bunker emissions, respectively. We also inferred the emissions from ROW (see Methods) where data are not directly available thus covering 100% of global CO_2_ emissions. Together, these data cover almost all fossil fuels and industry sources of global CO_2_ emissions, except for emissions from land use change (up to 10% of global CO_2_ emissions), and the non-fossil fuel CO_2_ emissions of industrial products (up to 2% of global CO_2_ emissions)^27^ and of cement and clinker (e.g., plate glass, ammonia, urea, calcium carbide, soda ash, ethylene, ferroalloys, alumina, lead and zinc).

**Fig. 2:**
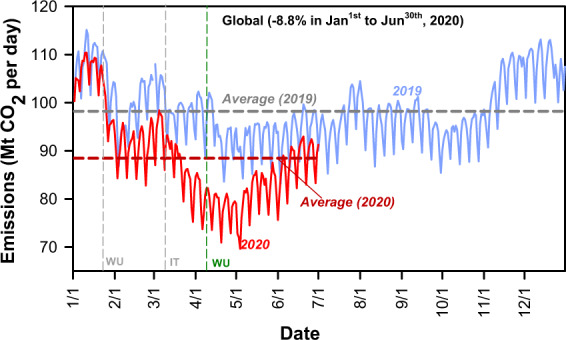
Daily CO_2_ emissions data from January 1^st^, 2019 to June 30^th^, 2020.

While daily emissions can be directly calculated using near-real-time activity data and emission factors for the electricity power sector, such an approach is difficult to apply to all sectors. For the industrial sector, emissions can be estimated monthly in some countries. For the other sectors, we used proxy data instead of daily real activity data to dynamically downscale the annual or monthly CO_2_ emissions totals to a daily basis. For instance, traffic indices in cities representative of each country were used instead of actual vehicle counts and categories, combined with annual national total sectoral emissions, to produce daily road transportation emissions. As such, for the use of fuels in the road transportation, air transportation and residential sectors in most countries, we downscaled monthly or annual total emissions data in 2019 to calculate the daily CO_2_ emissions in that year. Subsequently, we scaled monthly totals of 2019 by daily proxies of activities to obtain daily CO_2_ emissions data for the first four months of 2020 during the unprecedented disruption of the COVID-19 pandemic. The Carbon Monitor near-real-time CO_2_ emission dataset shows a 8.8% decline in CO_2_ emissions globally from January 1^st^ to June 30^th^ in 2020 when compared with the same period in 2019 (Fig. 2), and detects a regrowth of CO_2_ emissions by late April, which is mainly attributed to the recovery of economic activities in China and partial easing of lockdowns in other countries (for a more in-depth analysis of this topic, please see our recent related paper^28^).

## Methods

### Annual total and sectoral emissions per country in the baseline year 2019

According to the IPCC guidelines for emissions reporting^4^, the CO_2_ emissions *Emis* should be calculated by multiplying the activity data *AD* by the corresponding emissions factors *EF*

1 $$Emis=\sum \sum \sum A{D}_{i,j,k}\cdot E{F}_{i,j,k}$$

where *i*, *j*, *k* are indices for regions, sectors and fuel types, respectively. *EF* can be further separated into the net heating values *v* for each fuel type (the energy obtained per unit of fuel), the carbon content *c* per energy output (t C/TJ) and the oxidization rate *o* (the fraction (in %) of fuel oxidized during combustion):

2 $$Emis=\sum \sum \sum A{D}_{i,j,k}\cdot ({v}_{i,j,k}\cdot {c}_{i,j,k}\cdot {o}_{i,j,k})$$

Due to the lag of more than two years in the publishing governmental energy statistics, we started from the most recent CO_2_ emissions estimates up to 2018 from current CO_2_ databases^1,9–11^. For 2019, we completed this information by obtaining annual total emissions based on data in the literature and disaggregated the annual total into daily emissions (see below). For 2020, we estimated daily CO_2_ emissions by using daily changes in activity data in 2020 compared to 2019. The CO_2_ emissions and sectoral structure in 2018 for countries and regions were extracted from EDGAR V4.3.2^1^ and V5.0^29^ for each country, and national emissions were scaled to 2019 based on our own estimate (for China) and data from the Global Carbon Budget 2019^24^ (for other countries) (Table 1):

3 $$Emi{s}_{r,2019}={\alpha }_{r}\cdot Emi{s}_{r,2018}$$

**Table 1:** Scaling factor for the emission growth in 2019 compared to 2018.

Countries/Regions	Scaling Factor (%)	Source
China	2.8%	Estimated in this study
India	1.8%	Global Carbon Budget 2019^24^
US	2.4%	Carbon Brief, 2020^33^
EU27&UK	−3.9%	Carbon Brief, 2020^33^
Russia	0.5%	=ROW
Japan	0.5%	=ROW
Brazil	0.5%	=ROW
ROW	0.5%	Global Carbon Budget 2019^24^

For China, we first calculated CO_2_ emissions in 2018 based on the energy consumption by fuel type and for cement production in 2018 from the China Energy Statistical Yearbook^30^ and the National Bureau Statistics^31^, following Eq. 1. We projected the energy consumption in 2019 from the annual growth rates of coal, oil and gas reported by the Statistical Communiqué^31^ and applied China-specific emission factors^17^ to obtain the annual growth rate of emissions in 2019. We projected China’s CO_2_ emissions based on our previous studies on the country-specific emission factor data in China^17^ and past trends of China’s CO_2_ emissions^17,32^. Our projection (2.6%) is similar to the GCP revised projection^33^ (2.0%), and the slight difference falls into the uncertainty range of the both estimations. For the US and EU27&UK, we used updated emissions growth rates in 2019 reported by CarbonBrief^33^. For countries with no estimates for emissions growth rates in 2019, such as Russia, Japan and Brazil, we assumed that their growth rate was 0.5% based on the emission growth rate of the rest of the world^24^. As the result, our daily average CO_2_ estimates in 2019 (98.2 Mt CO_2_ per day) are lower than the daily average CO_2_ estimates in 2019 from GCP (around 100 Mt CO_2_ per day). The discrepancies mainly came from the difference between EDGAR and GCP databases, showing a 2.1% global difference between these two databases in 2018.

In this study, the EDGAR detailed sectors were aggregated into several larger sectors (*s*): power sector, industrial sector, transport sector (ground transport, aviation and shipping), and residential sector, and the percentage estimates for the various sectors are derived from the EDGAR database. This is consistent with the new activity data we used below to compute daily variations. We used the sectoral distribution in 2018 from EDGAR to infer the sectoral emissions in 2019 for each country/region (Eq. 4), assuming that the sectoral distribution remained unchanged in these two years.

4 $$Emi{s}_{r,s,2019}=Emi{s}_{r,2019}\cdot \frac{Emi{s}_{r,s,2018}}{Emi{s}_{r,2018}}$$

### Data acquisition and processing of carbon monitor daily CO_2_ emissions

According to IPCC Guidelines^4^, the CO_2_ emissions for each sector can be calculated by multiplying sectoral activity data by their corresponding emission factors following Eq. 5:

5 $$Emi{s}_{s}=A{D}_{s}\cdot E{F}_{s}$$

The emissions were calculated following this equation separately for the power sector, the industrial sector, the transport sector, and the residential sector.

### Power sector

The CO_2_ emissions from the power sector can be calculated by adapting Eq. 5 with sector-specific activity data (i.e., electricity production in Russia and thermal electricity production in other countries) and the corresponding emission factors (Eq. 6):

6 $$Emi{s}_{power}=A{D}_{power}\cdot E{F}_{power}$$

Normally, the emission factors change slightly over time but can be assumed to remain constant over the two-year period considered in this study compared to the large changes in activity data. We present uncertainties due to the changes of fuel mix in thermal production (see Technical Validation). Thus, we assumed that emission factors remained unchanged in 2019 and 2020 and calculated the daily emissions as follows:

7 $$Emi{s}_{daily}=Emi{s}_{yearly}\cdot \frac{A{D}_{daily}}{A{D}_{yearly}}$$

For China, we estimate the daily thermal production *AD*_*daily*_ from the daily disaggregation of monthly thermal emissions *AD*_*monthly*_ by using daily coal consumption by six power companies in China *C*_*daily*_ as follow:

8 $$A{D}_{daily}=A{D}_{monthly}\cdot \left({C}_{daily}/{C}_{monthly}\right)$$

The data sources of daily activity data in the power sector are described in Table 2. The countries/regions listed in Table 2 account for more than 74% of the total CO_2_ emissions in the power sector. For emissions from other countries (ROW), which are not listed in Table 2, we estimated the power sector emission changes in 2020 based on periods of the national lockdown. For daily emission changes for the ROW in 2019, we first assumed a linear relationship between daily global emissions and daily total emissions for the countries listed in Table 2. Then, we classified each country according to whether it adopted lockdown measures based on official reports. Based on daily emissions data for the power sector of the countries listed in Table 2, we calculated the average percent change *α* of power sector emissions across those countries during their lockdown periods, and used it to estimate the emission reduction of each country in the rest of the world during their specific national lockdowns (Eq. 9, where c denotes a country in ROW and d denotes day of 2020), and aggregated them into daily emissions for each ROW country.

9 $$Emi{s}_{c,d(c)}=\begin{array}{cc}Daily\,Mean\,Emi{s}_{c,2019}\times (1-\alpha ) & ,d(c)\in lockdown\,period\end{array}\,inc$$

**Table 2:** Data sources of activity data in the power sector.

Country/Region	Data source	Sectors included	Resolution
China	National Bureau of Statistics (https://data.stats.gov.cn/) / WIND (https://www.wind.com.cn/)	Thermal production /Daily coal consumption for 6 power companies	Monthly/daily
India	Power System Operation Corporation Limited (https://posoco.in/reports/daily-reports/)	Thermal production (summarizing the production of *Coal*, *Lignite*, and *Gas, Naphtha & Diesel*)	Daily
US	Energy Information Administration’s (EIA) Hourly Electric Grid Monitor (https://www.eia.gov/beta/electricity/gridmonitor/)	Thermal production (summarizing the production of *Coal, Petroleum*, and *Natural Gas*)	Hourly
EU27 & UK	ENTSO-E Transparent platform (https://transparency.entsoe.eu/dashboard/show)	Thermal production (summarizing the production of *Fossil.Brown. coal.Lignite, Fossil.Coal.derived.gas,Fossil.Gas, Fossil.Hard.coal, Fossil.Oil, Fossil.Oil.shale*, and *Fossil.Peat*)	Hourly
Russia	United Power System of Russia (http://www.so-ups.ru/index.php)	Total generation	Hourly
Japan	Organization for Cross-regional Coordination of Transmission Operators (OCCTO) (https://www.occto.or.jp/en/)	Thermal generation	Hourly
Brazil	Operator of the National Electricity System (http://www.ons.org.br/Paginas/)	Thermal production	Hourly

### Industrial sector: Industrial and cement production

While daily production data are not directly available for industrial and cement production, the monthly CO_2_ emissions from the industrial and cement production sectors can be calculated by using monthly statistics of industrial production and daily data of electricity generation to disaggregate the monthly CO_2_ emissions into daily values. This calculation assumes a linear relationship between daily electricity generation for industry and daily industry production data to compute daily industry production.

The emissions from industrial production during fossil fuel combustion were calculated by multiplying the activity data (i.e., fossil fuel consumption data in the industrial sector) by the corresponding emissions factors by the type of fuel. Due to limited data availability, we assumed a linear relationship between daily industrial production and industrial fossil fuel use, and the emission factors remained unchanged. Therefore, the monthly emissions in 2019 for a country/region can be calculated by the following equation:

10 $$Emi{s}_{montly,2019,r}=Emi{s}_{yearly,2019,r}\cdot \left({P}_{monthly,2019,r}/{P}_{yearly,2019,i,r}\right)$$

The emissions from cement production during the chemical process of calcination of calcite were also calculated with Eq. (10).

Specifically, for China, the emissions from the industrial sector were further divided into those for the steel, cement, chemical, and other industries (indicated by index *i*):

11 $$Emi{s}_{montly,2019,China}={\sum }^{E}mi{s}_{yearly,2019,i}\cdot \left({P}_{monthly,2019,i}/{P}_{yearly,2019,i}\right)$$

For the monthly emissions in 2020 for a country/region, we used the following equation:

12 $$Emi{s}_{montly,2020,r}=Emi{s}_{monthly,2019,r}\cdot \left({P}_{monthly,2020,r}/{P}_{monthly,2019,r}\right)$$

where *P* is the industrial production for different industrial sectors (in China) or the total industrial production index (in other countries), as listed in Table 3. In China’s case, the January and February estimates were combined, as individual monthly data were not reported by the sources listed in Table 3 for these two months. The monthly industrial emissions were disaggregated to daily emissions using daily electricity data, as explained above.

**Table 3:** Data sources for indust^prial production.

Country/Region	Sector	Data	Data source
China	Steel industry	Crude steel production	World Steel Association website (https://www.worldsteel.org/)
	Cement industry	Cement and clinker production	National Bureau of Statistics (http://www.stats.gov.cn/english/)
	Chemical industry	Sulfuric acid, caustic soda, soda ash, ethylene, chemical fertilizer, chemical pesticide, primary plastic and synthetic rubber	National Bureau of Statistics (http://www.stats.gov.cn/english/)
	Other industry	Crude iron ore, phosphate ore, salt, feed, refined edible vegetable oil, fresh and frozen meat, milk products, liquor, soft drinks, wine, beer, tobaccos, yarn, cloth, silk and woven fabric, machine-made paper and paperboards, plain glass, ten kinds of nonferrous metals, refined copper, lead, zinc, electrolyzed aluminum, industrial boilers, metal smelting equipment, and cement equipment	National Bureau of Statistics (http://www.stats.gov.cn/english/)
India	/	Industrial Production Index (IPI)	Ministry of Statistics and Programme Implementation (http://www.mospi.nic.in) Trading Economics (https://tradingeconomics.com)
US	/	Industrial Production Index (IPI)	Federal Reserve Board (https://www.federalreserve.gov)
EU & UK	/	Industrial Production Index (IPI)	Eurostat (https://ec.europa.eu/eurostat/home) Trading Economics (https://tradingeconomics.com)
Russia	/	Industrial Production Index (IPI)	Federal State Statistics Service (https://eng.gks.ru)
Japan	/	Industrial Production Index (IPI)	Ministry of Economy, Trade and Industry (https://www.meti.go.jp)
Brazil	/	Industrial Production Index (IPI)	Brazilian Institute of Geography and Statistics (https://www.ibge.gov.br/en/institutional/the-ibge.htm)

Lacking the latest Industrial Production Index for June 2020 for the EU27 & UK and India, we adopted monthly growth rates of industrial output from Trading Economics (https://tradingeconomics.com) based on preliminary survey data. CO_2_ emissions from countries listed in Table 3 accounts for more than 71% of the global industrial emissions. For other countries not listed in Table 3, we used the same method described for the power sector to calculate the daily industry emissions from the ROW.

To allocate monthly emissions into daily emissions, we assume the linear relationship between daily industry activity and daily electricity production, and use the weight of daily electricity production to monthly electricity production:

13 $$Emi{s}_{daily}=Emi{s}_{monthly}\cdot \left(Ele{c}_{daily}/Ele{c}_{monthly}\right)$$

### Transport sector

#### Ground transportation

We collected hourly congestion level data from the TomTom website (https://www.tomtom.com/en_gb/traffic-index/). The congestion level (hereafter called *X*) represents the extra time spent on a trip, as a percentage, compared to under uncongested conditions. TomTom congestion level data were obtained for 416 cities across 57 countries (Only-online Table 1) at a temporal resolution of one hour. Of note, a zero-congestion level means that the traffic is fluid or ‘normal’ but does not mean there are no vehicles and zero emissions. It is thus important to identify the lower threshold of emissions when the congestion level is zero. To do so, we compared the time series of daily mean TomTom congestion level *X* with the daily mean car flux (in vehicles per day) from publicly available real-time *Q* data from an average of 60 roads in the megacity area of Paris. The daily mean car counts were reported by the city’s service (https://opendata.paris.fr/pages/home/). We used a sigmoid function to fit the relationship between *X* and *Q*:

14 $$Q=a+\frac{b{X}^{c}}{{d}^{c}+{X}^{c}}$$

where a, b, c and d are the regression parameters (Table 4). We verified that the empirical fit from Eq. 14 can reproduce the observed large drop in *Q* due to the lockdown in Paris and the recovery afterwards. We assume that relative changes in daily emissions were proportional to the relative change in the function *Q*(*X*) from Eq. 14. Then, we applied the function *Q*(*X*) established for Paris to other cities included in the TomTom dataset, assuming that the relative magnitude of car counts (and thus emissions) follows a similar relationship with TomTom. The emission changes were first calculated for individual cities and then weighted by city emissions for aggregation to national changes. For a specific country *i* with *n* cities reported by TomTom, the national daily vehicle flux for day *j* was given by:

15 $${Q}_{country,dayj}=\frac{{\sum }_{i=1}^{n}{Q}_{i,dayj}{E}_{i}}{{\sum }_{i=1}^{n}{E}_{i}}$$

where *E*_*i*_ is the annual road transportation emissions of city *n* taken at the grid point of each TomTom city from the annual gridded EDGARv4.3.2 emissions map for the “road transportation” sector (1A3b) (https://edgar.jrc.ec.europa.eu/) for the year 2010, assuming that the spatial distribution of ground transport did not change significantly within a country between 2010 and the period of this study. Then, the daily road transportation emissions in 2019 and 2020 (${E}_{country,dayj}$) for a country were scaled such that the total road transportation emissions in the first half year of 2019 equaled 182/365 times the annual emissions of this sector in 2019 (${E}_{country,2019}$) estimated in this study:

16 $${E}_{country,dayj}={Q}_{country,dayj}\frac{182/365\times {E}_{country,2019}}{{\sum }_{j=1}^{182}{Q}_{country,dayj(2019)}}$$

**Table 4:** Regression parameters of the sigmoid function of Eq. 14 that describes the relationship between car counts (*Q*) and TomTom congestion level (*X*).

Parameter	Value
*a*	100.87
*b*	671.06
*c*	1.98
*d*	6.49

The TomTom GPS products include devices for car (https://www.tomtom.com/en_us/drive/car/), motorcycle (https://www.tomtom.com/en_us/drive/motorcycle/) and large vehicles (https://www.tomtom.com/en_us/drive/truck/). Although we did not find more information about the details how the congestion index is calculated, we believe that the calculation of congestion level includes the data from private and commercial cars, light and heavy vehicles. In this study, we did not compute the emissions separately for light and heavy vehicles separately, because 1) the EDGAR emission product for “road transport” (sector 1A3b), which we used as a reference emission product, did not separate these two different types of vehicles; and 2) the TomTom congestion level is not reported for light and heavy vehicles separately. So we implicitly assume that they similarly scale with the TomTom congestion level.

For countries not included in the TomTom dataset, we assumed that the emissions changes follow the mean changes of other countries. For example, Cyprus, as an EU member country, had no city reported in the TomTom dataset, so its relative emissions change was assumed to follow the same pattern for total emissions from other EU countries included in the TomTom dataset (which covers 98% of total EU emissions). Similarly, the relative changes in emissions for countries in the ROW but not reported by TomTom were assumed to follow the same pattern as the total emissions from all TomTom reported countries (which cover 85% of global total emissions).

#### Aviation

CO_2_ emissions from commercial aviation are usually reconstructed from bottom-up emission inventories based on knowledge of the parameters of individual flights^34,35^. We also calculated the CO_2_ emissions from commercial aviation following this approach. Individual commercial flights are tracked by Flightradar24 (https://www.flightradar24.com) based on ADS-B signals emitted by aircraft and received by their network of ADS-B receptors. As we do not yet have the capability to convert the FlightRadar24 database into CO_2_ emissions on a flight-by-flight basis, we compute CO_2_ emissions by assuming a constant *EF*_*aviation*_ (CO_2_ emission factor per km flown) across the whole fleet of aircraft (regional, narrowbody passenger, widebody passenger and freight operations). This assumption is reasonable if the flight mix between these categories has not changed significantly between 2019 and 2020.The International Council on Clean Transportation (ICCT) published that CO_2_ emissions from commercial freight and passenger aviation resulted in 918 Mt CO_2_ in 2018^36^ based on the OAG flight database and emission factors from the PIANO database. IATA estimated a 3.4% increase between 2018 and 2019 in terms of available seat kilometers^37^. In the absence of further information, we consider this increase to be representative of freight aviation as well and use a slightly smaller growth rate of 3% for CO_2_ emissions between 2018 and 2019 to account for a small increase in fuel efficiency. The kilometers flown are computed assuming great circle distance between the take-off, cruising, descent and landing points for each flight and are cumulated over all flights. The FlightRadar24 database has incomplete data for some flights and may completely miss a small fraction of actual flights, so we scale the ICCT estimate of CO_2_ emissions (inflated by 3% for 2019) with the total estimated number of kilometers flown for 2019 (67.91 million km) and apply this scaling factor to 2020 data. Again, this assumes that the fraction of missed flights is the same in 2019 and 2020. As the departure and landing airports are known for each flight, we can classify the km flown (and hence the CO_2_ emissions) per country and for each country between domestic or international traffic. The daily CO_2_ emissions were computed as the product of distance flown by a CO_2_ emission factor per km flown, according to:

17 $$Daily\,Emi{s}_{aviation}=Daily\,Kilometers\,Flow{n}_{aviation2020}\times E{F}_{aviation2019}$$

#### Ships

We collected international CO_2_ shipping emissions for 2016–2018 based on EDGAR’s international emissions. We also collected global shipping emissions during the period of 2007–2015 from IMO^38^ and ICCT^39^. According to the Third IMO GHG Study^38^, CO_2_ emissions from international shipping accounted for 88% of global shipping emissions, and domestic and fishing emissions accounted for 8% and 4%, respectively. We calculated international CO_2_ shipping emissions from 2007–2015 from global shipping emissions and the ratio of international to global shipping emissions. We extrapolated emissions from linear fits from 2007–2018 to estimate the emissions in 2019. The data sources of shipping emissions are listed in Table 5. We obtained emissions for the first half year of 2019 byassuming the equal distribution of monthly shipping CO_2_ emissions. The equations are as follows:

18 $$Monthly\,Emi{s}_{internationalshipping,2019}=\alpha \times Yearly\,Emi{s}_{internationalshipping,2019}\times {R}_{month}$$

**Table 5:** Data sources used to estimate ship emissions.

Shipping Emissions	Sources
Global shipping emissions (2007–2012)	IMO^38^
Global shipping emissions (2013–2015)	ICCT^39^
International shipping emissions (2016–2018)	EDGAR v5.0^13^

*α* is the increasing rate of international shipping emissions in 2019 based on the linear extrapolation of data from the period 2007–2018, estimated to be 3.01%. ${R}_{month}$ represents the ratio of the months to be calculated in the whole year. Given this, we estimated the shipping emissions for the first half year of 2019 using ${R}_{month}$ equal to 181/365.

We assumed that the change in shipping emissions was linearly related to the change in ship traffic volume. The change in international shipping emissions for the first half year of 2020 was calculated according to the following equation:

19 $$Emi{s}_{period,2020}=Emi{s}_{period,2019}\times {C}_{index}$$

where *c*_*index*_ represents the ratio of the change in shipping emissions, estimated to the end of April as −25% compared to the same period last year according to the news report^40^.

### Residential sector: residential and commercial buildings

Fuel consumption daily data from this sector are not available. Several studies^41,42^ showed that the main source of daily and monthly variability in this sector is climate, namely, heating emissions increase when temperature falls below a threshold that depends on the region, building type and people’s habits. We calculated emissions by assuming annual totals unchanged from 2019 and using daily climate information in three steps: 1) estimate population-weighted heating degree days for each country and for each day based on the ERA5^43^ reanalysis of 2-meter air temperature, 2) split residential emissions into two parts: cooking emissions and heating emissions based on the EDGAR database^29^ and using the EDGAR estimates of 2018 residential emissions as the baseline. Emissions from cooking were assumed to remain independent of temperature, and those from heating were assumed to be a function of the heating demand. Based on the change in population-weighted heating degree days in each country in 2019 and 2020, we downscaled annual EDGAR 2018 residential emissions to daily values for 2019 and 2020 as described by Eqs. 20–22:

20 $$Emi{s}_{c,m}=Emi{s}_{c,m,2018}\times \frac{\sum _{{\rm{m}}}HD{D}_{c,d}}{\sum _{{\rm{m,2018}}}HD{D}_{c,d}}$$

21 $$Emi{s}_{c,d}=Emi{s}_{c,m}\times Rati{o}_{heating,c,m}\times \frac{HD{D}_{c,d}}{\sum _{{\rm{m}}}HD{D}_{c,d}}+Emi{s}_{c,m}\times \left(1-Rati{o}_{heating,c,m}\right)\times \frac{1}{{N}_{m}}$$

22 $$HD{D}_{c,d}=\frac{\sum \left(Po{p}_{grid}\times ({T}_{grid,c,d}-18)\right)}{\sum \left(Po{p}_{grid}\right)}$$

where *c* is country, *d* is day, *m* is month, $Emi{s}_{c,m}$ is the residential emissions of country *c* in month *m* of year 2019 or 2020, $Emi{s}_{c,m,2018}$is the emissions of country *c* in month *m* of year 2018, $HD{D}_{c,d}$ is the population-weighted heating degree day in country *c* in day *d*, $Emi{s}_{c,d}$ is the residential emissions of country *c* in day *d* of year 2019 or 2020, $Rati{o}_{heating,c,m}$ is the percentage of residential emissions from heating demand in country *c* in month *m*, *N*_*m*_ is the number of days in month *m*, $Po{p}_{grid}$ is gridded population data derived from Gridded Population of the World, Version 4^44^, *T* is the daily average air temperature at 2 meters derived from ERA5^43^, and 18 is a HDD reference temperature^13^ of 18 °C.

**Fig. 3:**
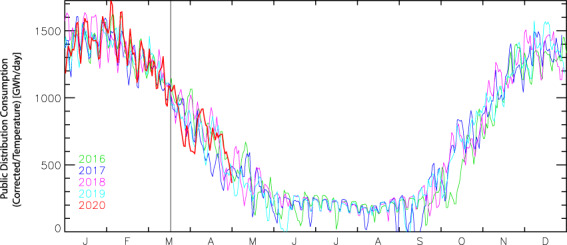
Residential and commercial building daily natural gas consumption (linearly related to CO_2_ emissions from this sector) in France for the last 5 years. Temperature effects have been removed from emissions using a linear piecewise model fitted to daily data. When the effect of variable winter temperature was removed, no significant change is seen in 2020 during the very strict lockdown period except for a small dip by end of March.

The main assumption in this approach is that residential emissions did not change based on factors other than heating degree day variations in 2020, although people’s time at home dramatically increased during the lockdown period. To test the validity of this assumption, we compiled natural gas daily consumption data by residential and commercial buildings for France (https://www.smart.grtgaz.com/fr/consommation) (unfortunately, such data could not be collected in many countries) during 2019 and 2020 (Fig. 3). Natural gas consumption in kWh per day was transformed to CO_2_ emissions using an emission factor of 10.55 kWh per m^3^ and a molar volume of 22.4 10^−3^ m^3^ per mole.

First, we verified that the temporal variation in those ‘true’ residential CO_2_ emissions was similar to that given by Eqs. 20–22. Second, after fitting a piecewise model to those natural gas residential emission data using ERA5 air temperature data, we removed the effect of temperature to obtain emissions corrected for temperature effects. Even if the lockdown was very strict in France, we found no significant emissions anomaly, meaning that although nearly the entire population was confined at home, it did not increase or decrease emissions. This complementary analysis tentatively suggests that residential emissions can be well approximated in other countries by Eqs. 20–22 based only on temperature during the lockdown period.

## Data Records

Currently, there are 36,177 data records provided in this dataset, which can be downloaded at our website (https://carbonmonitor.org) and Figshare^45^:

- A total of 270 records are daily mean CO_2_ emissions (from fossil fuel combustion and cement production processes) 1751–2020.
- A total of 4,923 records are the daily emissions for 9 countries/regions (i.e. China, India, US, EU27 & UK, Russia, Japan, Brazil, ROW and World) and 547 days (from January 1, 2019 to June 30^th^, 2020).
- A total of 3,829 records are the daily emissions for 6 sectors and the global total (i.e. power, industry, residential, ground transportation, aviation (including domestic aviation and international aviation), international shipping and Globe) and 547 days (from January 1, 2019 to June 30^th^, 2020).
- A total of 27,155 records are daily emissions of 9 countries/regions (i.e. China, India, US, EU27 & UK, Russia, Japan, Brazil, ROW and World) in the power sector and industrial sector and the global total in the international shipping sector for 547 days (from January 1, 2019, to June 30^th^, 2020), and ground transport sector, residential sector and aviation sector (China, India, US, EU27 & UK, Russia, Japan, Brazil, ROW, domestic aviation total, international aviation, and total aviation) for 578 days (from January 1, 2019, to July 31^st^, 2020).

## Technical Validation

### Uncertainty estimates

We calculate daily emissions directly from daily activity data in most sectors, thus the daily uncertainties are explained by the specific uncertainty of daily sectoral activity data itself and uncertainties in the (empirical) models used to convert activity to emissions. Here we should distinguish between *error* and *uncertainty*. Errors being defined the difference to the truth cannot be estimated because we do not know the true values. Uncertainty could be estimated if we had different activity datasets, by looking at the spread of these different datasets, but this is also not possible as most of our activity data are unique. The main issue is that we do not know if the uncertainty of activity data would be a systematic uncertainty (e.g. all days are biased low using our activity dataset compared to another activity dataset) or a random error (in which case, there could be positive bias one day compensated by negative bias another day and the uncertainty would be small e.g. on monthly values derived from daily values). We do acknowledge that we cannot estimate daily uncertainties. In the future, this could be done by trying to collect different activity data. This uncertainty analysis was also presented in our related paper recently published at *Nature Communications*^28^.

Thus, we followed the 2006 IPCC Guidelines for National Greenhouse Gas Inventories to conduct an uncertainty analysis of the data. First, the uncertainties were calculated for each sector (See Table 6 for uncertainty ranges of each sector):

- Power sector: the uncertainty is mainly from inter-annual variability of coal emission factors and changes in mix of generation fuel in thermal production. The uncertainty of power emission from fossil fuel is within (±14%) with the consideration of both inter-annual variability of fossil fuel based on the UN statistics and the variability of the mix of generation fuel (the ratio of electricity produced by coal to thermal production).
- Industrial sector: The uncertainty of CO_2_ from industry and cement production comes from monthly production data. CO_2_ from industry and cement production in China accounts for more than 60% of world total industrial CO_2_, and the uncertainty of emissions in China is 20%. Uncertainty from monthly statistics was derived from 10,000 Monte Carlo simulations to estimate a 68% confidence interval (1 sigma) for China. We calculated the 68% prediction interval of the linear regression models between emissions estimated from monthly statistics and official emissions obtained from annual statistics at the end of each year to deduce the one-sigma uncertainty involved when using monthly data to represent the change for the whole year. The squared correlation coefficients are within the range of 0.88 (e.g., coal production) and 0.98 (e.g., energy import and export data), which indicates that only using the monthly data can explain 88% to 98% of the whole year’s variation^32^; the remaining variation is not covered but reflects the uncertainty caused by the frequent revisions of China’s statistical data after they are first published.
- Ground Transportation: The emissions from the ground transportation sector are estimated by assuming that the relative magnitude in car counts (and thus emissions) follow a similar relationship with TomTom congestion index in Paris. This model calibrated in Paris was cross checked to match traffic fluxes of two other cities. Future work will focus on additional cross-validation and calibration with more traffic data from other cities.
- Aviation: The uncertainty in the aviation sector comes from the difference in daily emission data estimated based on the two methods. We calculate the average difference between the daily emission results estimated based on the flight route distance and the number of flights and then divide the average difference by the average daily emissions estimated by the two methods to obtain the uncertainty in CO_2_ from the aviation sector.
- Shipping: We used the uncertainty analysis from IMO as our uncertainty estimate for shipping emissions. According to the Third IMO Greenhouse Gas study 2014^38^, the uncertainty in shipping emissions was 13% based on bottom-up estimates.
- Residential: The 2-sigma uncertainty in daily emissions is estimated as 40%, which is calculated based on a comparison with daily residential emissions derived from real fuel consumption in several European countries, including France, Great Britain, Italy, Belgium, and Spain.

**Table 6:** Percentage uncertainty of all items.

Items	Uncertainty Range
Power	±14.0%
Ground transport	±9.3%
Industry	±36.0%
Residential	±40.0%
Aviation	±10.2%
International shipping	±13.0%
Projection of emissions growth rate in 2019	±0.8%
EDGAR emissions in 2018	±5.0%
**Overall**	±**7.2%**

The uncertainty in the emission projection for 2019 is estimated as 2.2% by combining the reported uncertainty of the projected growth rates and the EDGAR estimates in 2018.

Then, we combine all the uncertainties by following the error propagation equation from the IPCC. Equation 23 is used to derive the uncertainty of the sum and could be used to combine the uncertainties of all sectors:

23 $${U}_{total}=\frac{\sqrt{\sum ({U}_{s}\cdot {\mu }_{s})}}{|\sum {\mu }_{s}|}$$

where ${U}_{s}$ and ${\mu }_{s}$ are the percentage and quantity (daily mean emissions) of the uncertainty of sector *s*, respectively.

Equation 24 is used to derive the uncertainty of the multiplication, which in turn is used to combine the uncertainties of all sectors and of the projected emissions in 2019:

24 $${U}_{overall}=\sqrt{\sum {U}_{i}^{2}}$$

## Data Availability

The generated datasets are available from 10.6084/m9.figshare.12685937.v4 and https://github.com/zhudeng94/dailyCO2. Codes for industrial emission calculation and summary table generation are available on the GitHub repository presented as worksheets. Also the raw data of power generation in U.S., EU27 & UK, India, Russia, Japan and Brazil are available on the GitHub repository. Other raw data and codes for emission estimation in other sectors are only available upon reasonable requests.

## Online-only Table

**Online-only Table 1:** Cities (416 across 57 countries) where TomTom congestion level data are available.

**Country/Region**	**City**
Austria (5)	Vienna, Salzburg, Graz, Innsbruck, Linz
Belgium (10)	Brussels, Antwerp, Namur, Leuven, Ghent, Liege, Kortrijk, Mons, Bruges, Charleroi
Bulgaria (1)	Sofia
Czech (3)	Brno, Prague, Ostrava
Denmark (3)	Copenhagen, Aarhus, Odense
Estonia (1)	Tallinn
Finland (3)	Helsinki, Turku, Tampere
France (25)	Paris, Marseille, Bordeaux, Nice, Grenoble, Lyon, Toulon, Toulouse, Montpellier, Nantes, Strasbourg, Lille, Clermont-Ferrand, Brest, Rennes, Rouen, Le Havre, Saint Etienne, Nancy, Avignon, Orleans, Le Mans, Dijon, Reims, Tours
Germany (26)	Hamburg, Berlin, Nuremberg, Bremen, Stuttgart, Munich, Bonn, Frankfurt am main, Dresden, Cologne, Wiesbaden, Ruhr Region West, Leipzig, Hannover, Kiel, Freiburg, Dusseldorf, Karlsruhe, Ruhr Region East, Munster, Augsburg, Monchengladbach, Mannheim, Bielefeld, Wuppertal, Kassel
Greece (2)	Athens, Thessaloniki
Hungary (1)	Budapest
Iceland (1)	Reykjavik
Ireland (3)	Dublin, Cork, Limerick
Italy (25)	Rome, Palermo, Messina, Genoa, Naples, Milan, Catania, Bari, Reggio Calabria, Bologna, Florence, Turin, Prato, Cagliari, Pescara, Livorno, Trieste, Verona, Taranto, Reggio Emilia, Ravenna, Padua, Parma, Modena, Brescia
Latvia (1)	Riga
Lithuania (1)	Vilnius
Luxembourg (1)	Luxembourg
Netherlands (17)	The Hague, Haarlem, Leiden, Arnhem, Amsterdam, Rotterdam, Nijmegen, Groningen, Eindhoven, Utrecht, Amersfoort, Tilburg, Breda, Apeldoorn, Zwolle, Den Bosch, Almere
Norway (4)	Oslo, Trondheim, Stavanger, Bergen
Poland (12)	Lodz, Krakow, Poznan, Warsaw, Wroclaw, Bydgoszcz, Gdansk-Gdynia-Sopot, Szczecin, Lublin, Bialystok, Bielsko-Biala, Katowice urban area
Portugal (5)	Lisbon, Porto, Funchal, Braga, Coimbra
Romania (1)	Bucharest
Russia (11)	Moscow, Saint Petersburg, Novosibirsk, Yekaterinburg, Nizhny Novgorod, Samara, Rostov-on-don, Chelyabinsk, Omsk, Tomsk, Kazan
Slovakia (2)	Bratislava, Kosice
Slovenia (1)	Ljubljana
Spain (25)	Barcelona, Palma de Mallorca, Granada, Madrid, Santa Cruz de Tenerife, Seville, A Coruna, Valencia, Malaga, Murcia, Las Palmas, Alicante, Santander, Pamplona, Gijon, Cordoba, Zaragoza, Vitoria Gasteiz, Vigo, Cartagena, Valladolid, Bilbao, Oviedo, San Sebastian, Cadiz
Sweden (4)	Stockholm, Uppsala, Gothenburg, Malmo
Switzerland (6)	Geneva, Zurich, Lugano, Lausanne, Basel, Bern
Turkey (10)	Istanbul, Ankara, Izmir, Antalya, Bursa, Adana, Mersin, Gaziantep, Konya, Kayseri
Ukraine (4)	Kiev, Odessa, Kharkiv, Dnipro
UK (25)	Edinburgh, London, Bournemouth, Hull, Belfast, Brighton and Hove, Bristol, Manchester, Leicester, Coventry, Nottingham, Cardiff, Birmingham, Southampton, Leeds-Bradford, Liverpool, Sheffield, Swansea, Newcastle-Sunderland, Glasgow, Reading, Portsmouth, Stoke-on-Trent, Preston, Middlesbrough
Egypt (1)	Cairo
South Africa (6)	Cape Town, Johannesburg, Pretoria, East London, Durban, Bloemfontein
China (22)	Chongqing, Zhuhai, Guangzhou, Beijing, Chengdu, Changchun, Changsha, Shenzhen, Shenyang, Shanghai, Wuhan, Fuzhou, Shijiazhuang, Xiamen, Nanjing, Hangzhou, Tianjin, Ningbo, Quanzhou, Dongguan, Suzhou, Wuxi
Hong Kong (1)	Hong Kong
India (4)	Mumbai, New Delhi, Bangalore, Pune
Indonesia (1)	Jakarta
Israel (1)	Tel Aviv
Japan (5)	Tokyo, Osaka, Nagoya, Sapporo, Kobe
Kuwait (1)	Kuwait City
Malaysia (1)	Kuala Lumpur
Philippines (1)	Manila
Saudi Arabia (2)	Riyadh, Jeddah
Singapore (1)	Singapore
Taiwan (5)	Kaohsiung, Taipei, Taichung, Tainan, Taoyuan
Thailand (1)	Bangkok
United Arab Emirates (2)	Dubai, Abu Dhabi
Australia (10)	Sydney, Melbourne, Brisbane, Adelaide, Gold Coast, Hobart, Newcastle, Perth, Canberra, Wollongong
New Zealand (6)	Auckland, Wellington, Hamilton, Christchurch, Dunedin, Tauranga
Argentina (1)	Buenos Aires
Brazil (9)	Recife, Sao Paulo, Rio de Janeiro, Salvador, Fortaleza, Porto Alegre, Belo Horizonte, Curitiba, Brasilia
Chile (1)	Santiago
Columbia (1)	Bogota
Peru (1)	Lima
Canada (12)	Vancouver, Toronto, Montreal, Ottawa, London, Winnipeg, Halifax, Quebec, Hamilton, Calgary, Edmonton, Kitchener-Waterloo
Mexico (1)	Mexico City
USA (80)	Los Angeles, New York, San Francisco, San Jose, Seattle, Miami, Chicago, Washington, Honolulu, Atlanta, Baton Rouge, San Diego, Boston, Austin, Portland, Philadelphia, Sacramento, Houston, Riverside, Tampa, Nashville, Orlando, Charleston, Denver, Cape Coral Fort Myers, Pittsburgh, New Orleans, Las Vegas, Boise, Fresno, Baltimore, Tucson, Providence, Charlotte, Dallas Fort Worth, Oxnard Thousand Oaks Ventura, Bakersfield, Greenville, Jacksonville, Detroit, Albuquerque, Columbus, San Antonio, Salt Lake City, Phoenix, McAllen, Raleigh, Virginia Beach, Hartford, Colorado Springs, Birmingham, New Haven, Louisville, Minneapolis, Cincinnati, El Paso, Allentown, Buffalo, Memphis, Worcester, Grand Rapids, Albany, St Louis, Milwaukee, Omaha Council Bluffs, Indianapolis, Rochester, Columbia, Oklahoma City, Cleveland, Tulsa, Kansas City, Knoxville, Richmond, Winston-Salem, Dayton, Little Rock, Syracuse, Akron, Greensboro-High Point
